# Added flavors: potential contributors to body weight gain and obesity?

**DOI:** 10.1186/s12916-022-02619-3

**Published:** 2022-11-01

**Authors:** Nathalie Judith Neumann, Mathias Fasshauer

**Affiliations:** 1 Institute of Nutritional Science, Justus-Liebig-University of Giessen, 35390 Giessen, Germany; 2 Department of Internal Medicine (Endocrinology, Nephrology, and Rheumatology), University of Leipzig, Leipzig, Germany

**Keywords:** Added flavors, Body weight, Flavor-nutrient learning, Food intake, Hedonic eating, Metabolic syndrome, Obesity, Ultra-processed food

## Abstract

**Background:**

Added flavors are a marker for ultra-processing of food and a strong link exists between the intake of ultra-processed food and the development of obesity. The objective of the present article is to assess animal and human data elucidating the impact of added flavors on the regulation of food intake and body weight gain, as well as to define areas for future research.

**Main text:**

Mechanistic studies suggest that added flavors induce overeating and body weight gain by two independent mechanisms: Added flavors promote hedonic eating and override homeostatic control of food intake, as well as disrupt flavor-nutrient learning and impair the ability to predict nutrients in food items. Supporting these potential mechanisms, added flavors increase feed intake and body weight as compared to non-flavored control diets in a broad range of animal studies. They are actively promoted by feed additive manufacturers as useful tools to improve palatability, feed intake, and performance parameters. In humans, added flavors are extensively tested concerning toxicity; however, no data exist concerning their impact on food intake and body weight.

**Conclusions:**

Added flavors are potential contributors to the obesity epidemic and further studies focusing on their role in humans are urgently required. These studies include obesity interventions specifically targeting food items with added flavors and cohort studies on independent associations between added flavor intake and metabolic, as well as cardiovascular, morbidity, and mortality.

**Supplementary Information:**

The online version contains supplementary material available at 10.1186/s12916-022-02619-3.

## Background

Food additives are substances added intentionally to food to preserve flavor, as well as to enhance taste, appearance, or other sensory qualities [1]. Examples for food additives are flavors, flavor enhancers, sweeteners, colors, emulsifiers, stabilizers, gelling agents, thickeners, and preservatives [1]. Added flavors, also called flavorings, are defined as products “not intended to be consumed as such, which are added to food in order to impart or modify odour and/or taste” [2]. The European food law distinguishes different categories of which added flavors can consist, such as flavor preparations which are obtained from different natural sources and flavor substances which are single chemical compounds [2]. Flavor substances can be called natural if they are “obtained by appropriate physical, enzymatic or microbiological processes from material of vegetable, animal or microbiological origin […] [and if they] correspond to substances that are naturally present and have been identified in nature” [2]. In the United States of America (US), artificial flavors are defined as substances not obtained from spices, fruits, vegetables, meat, or other natural sources, while natural flavors are essential oils, oleoresins, extractives, and other products derived from natural sources [3].

Added flavors are used in food for different reasons. Within the last decades, industrial processing of food has led to flavor losses which were compensated by added flavors [4, 5]. They save costs; improve, change, enhance, or complement the taste of products; and mask undesirable flavor characteristics [5, 6]. Added flavors equalize the taste of products with less sugar, fat, and salt whose demand is rising [7]. They are able to maintain the typical taste of products and, thus, meet consumer expectations even if there are variations in the raw materials [6, 7]. In the past, added flavors also masked spoilage and enabled the consumption of food that would otherwise have been thrown away [5]. Despite their frequent use in food, limited data are available on how added flavors influence excessive calorie intake and body weight.

In the present opinion article, arguments are presented that added flavors contribute to the obesity epidemic in recent decades. Thus, mechanistic studies suggest that added flavors induce overeating and body weight gain by two independent mechanisms: Added flavors promote hedonic eating and override homeostatic control of food intake, as well as disrupt flavor-nutrient learning and impair the ability to predict nutrients in food items. Supporting these potential mechanisms, added flavors increase feed intake, as well as body weight, in animals and are actively promoted by feed additive manufacturers. In humans, added flavors are extensively tested concerning toxicity; however, no data exist concerning their impact on food intake and body weight.

These arguments are based on the literature search summarized in Additional file 1 [8–13] and explored in more detail within the next chapters.

## Main text

### Potential mechanisms for flavor-induced weight gain

There are two potential mechanisms by which added flavors might induce food intake and body weight gain:

- Added flavors promote hedonic eating
- Added flavors disrupt flavor-nutrient learning

#### Promotion of hedonic eating

**Fig. 1:**
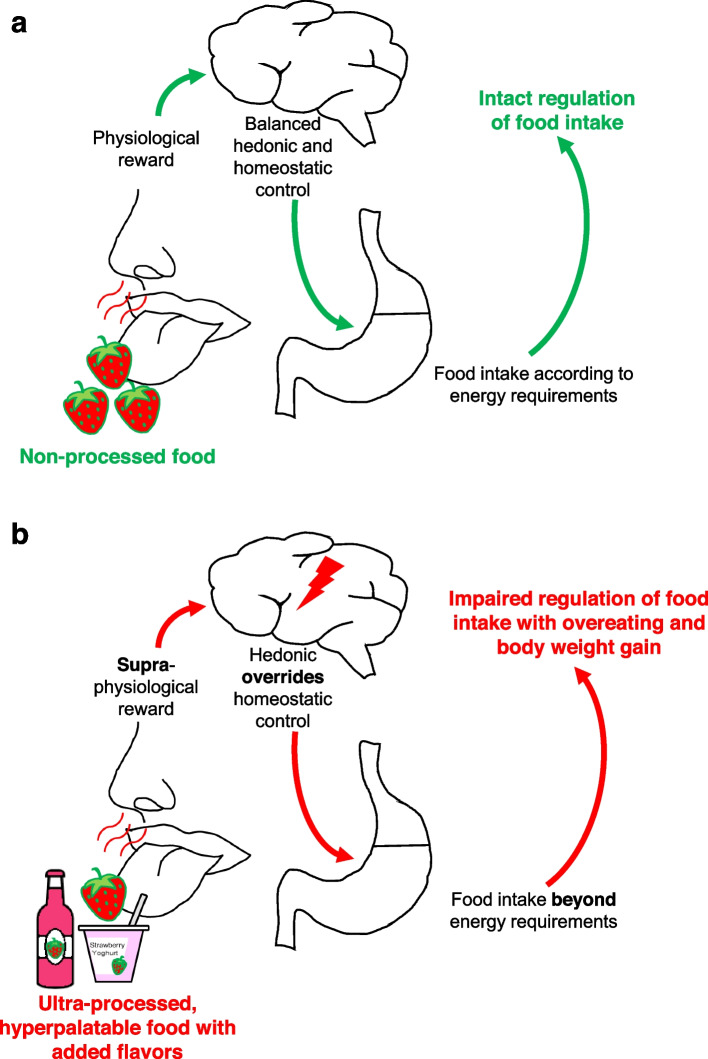
Promotion of hedonic eating. Simplified presentation of hedonic eating **a** being balanced with homeostatic control (example: fresh strawberries) and **b** overriding homeostatic control (example: strawberry-flavored food items)

Added flavors might induce overeating and weight gain by promoting hedonic eating and overriding homeostatic control of food intake (Fig. 1). Food intake and body weight are controlled by the homeostatic and the hedonic systems [14, 15]. The homeostatic control aims to maintain current body weight through metabolic regulation of food intake and energy expenditure, i.e., its primary goal is eating for survival [14, 15]. Hedonic eating, in contrast, is driven by the reward system and independent of energy balance, i.e., its primary aim is eating for pleasure [14, 15]. Food intake due to hedonic mechanisms may involve extra calories which would not have been consumed under homeostatic control [16]. In situations of energy deficiency, both systems work together to induce food intake and cover energy needs; however, they might collide in food-rich environments [16]. Processing increases rewarding properties and hedonic value of products compared to unprocessed food items [17, 18]. The availability of these palatable, energy-dense foods in the modern environment promotes hedonic pathways [14, 19, 20]. In response to those rewarding food items, the hedonic system is able to override homeostatic control despite energetically unbalanced conditions [14, 15, 20]. This “eating in the absence of hunger” (EAH) was convincingly shown in adolescents who ate highly palatable snacks even after a meal that exceeded energy requirements [21]. EAH has also been linked to weight gain, food overconsumption, and loss of control over eating in adults [22–24]. Permanent hedonic overeating can cause weight gain in the long term [14]. Added flavors and other so-called cosmetic additives make products palatable or even hyperpalatable [25, 26]. Therefore, added flavors might increase rewarding characteristics of food, promote hedonic eating, and override homeostatic control of food intake, leading to obesity in the long term. This potential mechanism is illustrated in Fig. 1. Scenario a depicts balanced regulation of food intake by the homeostatic and hedonic systems when exposed to non-processed food, e.g., fresh strawberries, as a physiological reward. Here, food is consumed according to energy requirements (Fig. 1a). In scenario b, the hedonic system overrides homeostatic control of food intake when exposed to ultra-processed, hyperpalatable food with added flavors as a supra-physiological reward, e.g., strawberry-flavored food items. Here, food is consumed for pleasure independent of energy requirements (Fig. 1b). It is interesting to note in this context that several additives including flavors, flavor enhancers, sweeteners, colors, and emulsifiers are markers for food ultra-processing according to the NOVA system which classifies products based on “nature, extent and purpose of the industrial processing they undergo” [25, 26]. A strong link exists between the intake of ultra-processed food and the development of obesity [27–30]. There is considerable data suggesting that processing techniques applied in the manufacture of ultra-processed food such as the deconstruction of the original food matrix structure, as well as the use of high amounts of sugar, salt, and fat enhance orosensory properties and energy density [25, 26]. As a consequence, eating rate is increased and endogenous satiety overridden, thereby, resulting in greater overall food intake [25, 26]. However, no study so far has analyzed the potential contribution of added flavors to excessive calorie intake and body weight gain in humans.

#### Disruption of flavor-nutrient learning

**Fig. 2:**
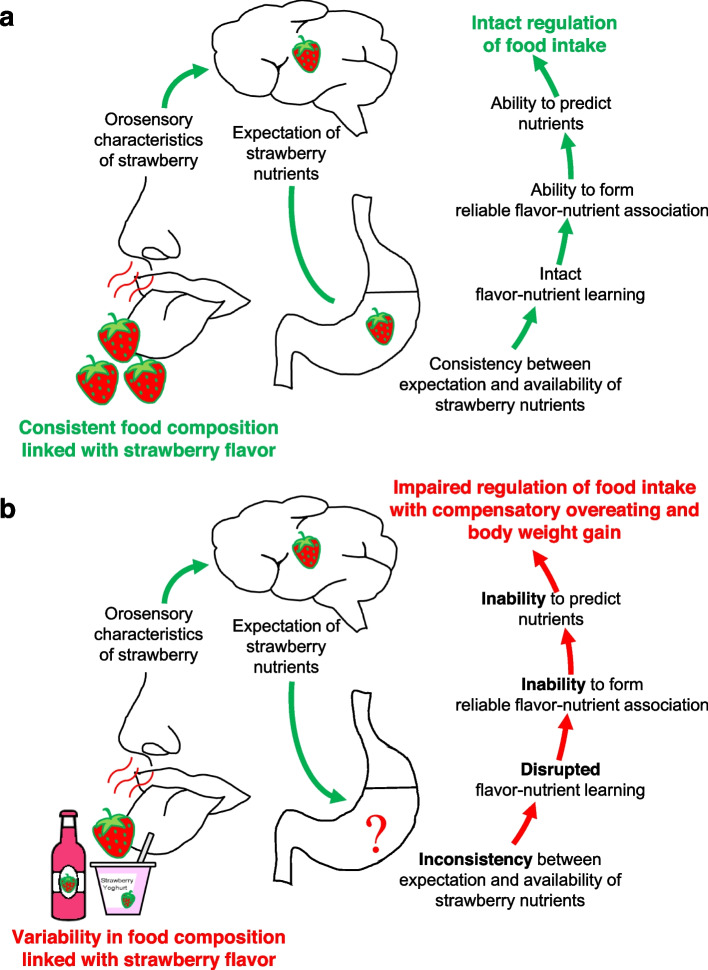
Disruption of flavor-nutrient learning. Simplified presentation of (**a**) intact flavor-nutrient learning (example: fresh strawberries) and (**b**) disrupted flavor-nutrient learning (example: strawberry-flavored food items)

Added flavors might induce overeating and weight gain by disrupting flavor-nutrient learning and impairing the ability to predict nutrients in food items (Fig. 2). Flavor-nutrient learning describes the process of developing flavor-nutrient associations through repeated experiences with the orosensory characteristics of a food and the subsequent physiological impacts and postingestive consequences [31, 32]. Due to these learned associations, food intake can be matched to nutritional needs (Fig. 2a) [33, 34]. Figure 2a provides a simplified overview of intact flavor-nutrient learning using the example of fresh strawberries. Their orosensory characteristics are always linked with nutrients of real strawberries (Fig. 2a). This consistency between expected and available nutrients promotes an intact flavor-nutrient learning which allows the formation of reliable flavor-nutrient associations and correct predictions of nutrients in the future with subsequent intact regulation of food intake (Fig. 2a).

Added flavors potentially cause inconsistency between orosensory characteristics of a meal and associated nutrients. Products might taste similar despite differences in their nutritional composition which causes a higher variability in food composition linked with a specific flavor. Convincing evidence suggests that variability leads to disruption of flavor-nutrient learning which subsequently impairs both formation of reliable flavor-nutrient associations and proper regulation of food intake causing overeating and subsequent body weight gain [32].

Figure 2b provides a simplified overview of disrupted flavor-nutrient learning using the example of strawberry-flavored food items besides fresh strawberries. These products have a similar taste despite differences in energy and nutrient content inducing inconsistency between expected and available strawberry nutrients which causes disruption of flavor-nutrient learning (Fig. 2b). As a consequence, the inability to correctly predict nutrients leads to compensatory overeating and body weight gain (Fig. 2b). It is interesting to note in this context that rats exposed to inconsistent flavor-calorie pairings gained about 7% more weight than control rats exposed to a consistent flavor-calorie relationship [33]. Similarly, humans familiar with a wide variability of pizzas with different energy content have a lower expected satiation of a pizza and a decreased ability to compensate calories of eaten pizza in a subsequent test meal [35].

### Added flavors in animal feed

Added flavors have been used in the production of animal feed for more than 40 years [36]. In pigs, they are applied in the weaning period in combination with synthetic sweeteners [37]. Palatant additives like added flavors are also included in a wide range of ruminant feed, e.g., milk replacers, mineral premix, compound, and concentrated feed [38]. The use of added flavors in calves is approved and recognized as absolutely necessary [37]. Although there is evidence that the feed intake can also be increased in cattle, the higher costs for flavored feed might make the use of added flavors unattractive to farmers [37]. Added flavors are also common additives for reward items in horses [37].

Added flavors are actively promoted by feed additive manufacturers. One manufacturer states that its flavors “are used by some of the biggest feed companies in the world” [39]. The same manufacturer suggests that “feeding pigs with a well-balanced diet that is highly palatable is essential for optimal growth performance and production efficiency” and that “flavors are useful tools to improve palatability and feed intake” [40]. Another manufacturer states that “addition of flavors to ruminant diets is a useful tool to improve palatability, increase feed intake and performance parameters” [38].

Various studies have assessed the impact of added flavors on feed intake, as well as body weight, in animals and the main results are summarized below and in Additional file 2. Furthermore, the species used, period of life, duration of intervention period, and added flavor tested are presented for each study in Additional file 2. There is a large diversity in reports assessing the use of added flavors in animal nutrition. In general, three different study designs and endpoints can be distinguished:

1. The first kind of studies tested the preference of animals regarding flavored and non-flavored feed. The same animals were exposed at the same time period to one or more feeds with added flavors (intervention) and a non-flavored feed (control). Feed intake per time unit was the primary outcome in these experiments. It was significantly higher for at least one added flavor tested in the intervention as compared to the control in studies in goats [41] and ponies [42]. However, one preference study did not find differences between flavored and non-flavored feed in lactating cows [43] and another study even described a significantly lower intake of flavored feed in post-weaning piglets [44].
2. In a second type of studies using a within-subject design, the same animals were exposed consecutively to one or more feeds with added flavors (intervention) and non-flavored feed (control). Feed intake and body weight gain per time interval were the primary outcomes in these experiments. They were higher in the intervention as compared to the control for orange flavor in a study with calves but no difference was found in the feed intake of second-lactation cows [45]. In another study in baboons, there was a trend towards higher feed intake for one (chocolate, fruit punch, lemon, orange) but not another (apple, lemon, orange, sugar) set of added flavors [46]. In a second experiment, consecutive application of a punch and orange flavor increased the feed intake [46]. The authors concluded that the results “may be useful for producing a nonhuman primate model of obesity” [46]. However, the study had two major drawbacks which might bias the findings: First, the proportion of simple carbohydrates was different between flavored (25%) and unflavored (5%) chow. Second, the unflavored feed was also offered during the intervention period.
3. In a third kind of studies, applying a between-subject design, groups of different animals were exposed to either one or more feeds with added flavors (intervention) or non-flavored feed (control). Comparisons of feed intake, body weight gain, and final body weight between the intervention and control groups were the primary outcomes in these experiments. Feed intake was significantly higher in the intervention as compared to the control for at least one added flavor in various studies in pre-weaning piglets [47–50], post-weaning piglets [47, 49, 51], lactating sows [52], and pre-weaning calves [53], and there was a trend towards higher feed intake in pre-weaning and post-weaning piglets [47], as well as post-weaning calves [54]. Body weight gain was significantly higher in the intervention as compared to the control for at least one added flavor in several reports in pre-weaning piglets [52], post-weaning piglets, and growing pigs [47–50, 55, 56], as well as in pre-weaning calves [53, 54] and in calves generally [57]. There was a trend towards higher body weight gain in post-weaning piglets [51] and post-weaning calves [54]. Furthermore, final body weight was significantly higher in the intervention as compared to the control group in pre-weaning piglets [47, 52], post-weaning piglets [47, 55], and pre-weaning and post-weaning calves [53]. In some reports, body weight tended to be higher in post-weaning piglets [50] and in calves [57]. However, several studies did not observe differences between the intervention and control groups in at least one period of life concerning feed intake [44, 48, 50, 51, 53–62], body weight gain [44, 47–49, 51–53, 58–61], and final body weight [44, 49, 50, 52, 56, 58–60]. One study shows convincingly that novel flavors unconditionally suppress weight gain but not feed intake in the absence of flavor-calorie associations in rats [62].

Combined, these studies indicate that added flavors can increase feed intake, body weight gain, and final body weight. However, none of these animal studies focused on the mechanisms for these effects. Therefore, additional studies should assess how promotion of hedonic eating, disruption of flavor-nutrient learning, and other potential mechanisms contribute to increased feed intake, body weight gain, and final body weight. Moreover, results were heterogeneous and changes in feed intake, body weight gain, and final body weight were not always in the same direction. A major drawback is that several between-subject design studies did not assess all three endpoints (Additional file 2). Furthermore, flavor preference cannot be elucidated in animals directly, e.g., with consumer sensory evaluation as in humans [63], but only indirectly by measuring differences in feed intake. It needs to be elucidated in future studies how these data in animals related to flavored versus non-flavored diets can be translated to human obesity.

### Use of added flavors in human nutrition

Within the European Union, flavor substances must be approved before they can be added to food in human nutrition [2]. Their safety is evaluated by the European Food Safety Authority and approved flavor substances are listed in a positive list in Annex I of regulation 1334/2008 [2, 64]. In the US, the Food Additives Amendment from 1958 distinguishes between food additives which have to be approved by the Food and Drug Administration and substances graded as “generally recognized as safe” by qualified experts [65–67]. Nevertheless, approval of added flavors within the European Union and the US does not require testing concerning endpoints like body weight gain and no such studies in humans have been published to the best of our knowledge.

The use of added flavors in human nutrition reaches far back in the past with monks in the medieval age already using natural substances obtained by distillation from plant material to flavor food [68]. The industrial production of flavors started in the nineteenth century with the first flavor compounds being isolated from natural sources [69]. Soon, chemical synthesis of flavor substances started, e.g., the production of vanillin synthesized by Tiemann and Haarmann in 1874 [69, 70]. The production of flavors and fragrances has been closely linked within one industry, called the flavor and fragrance industry, with almost equal market shares of the flavor and fragrance parts [71]. In the twentieth century, some decisive changes advanced the flavor and fragrance industry. These include the discovery of spray-drying flavors in 1930 [72] and the invention of gas chromatography in the 1960s which led to further exploration and discovery of flavors [73, 74]. Around the same time, there was a trend towards a healthier lifestyle and consumers attached importance to the naturalness of their food [75, 76]. This led to a shift from synthetic to natural flavors and was a challenge for the flavor and fragrance industry which at that time was focused on the development of new synthetic flavors [75]. Due to this increasing consumer demand, natural flavors in flavored food novelties increased from about 35% in 1965 to 80% in 1995 with a concomitant decrease in prices for naturals [75].

In the second half of the twentieth century, the worldwide flavor and fragrance industry expanded tremendously. For the US flavor and fragrance industry, sales adjusted for 2020 US$ value increased from US$ 1.9 billion in 1963 to US$ 7.0 billion in 2013 (Additional file 3) [77–90]. In parallel to the growth of the flavor and fragrance industry, there was an increase in US obesity prevalence from 13.4% in 1961 to 38.2% in 2013/2014 (Additional file 3) [91]. The parallel increase in obesity rates and flavor and fragrance industry sales does not serve as a proof in itself for weight-inducing effects of added flavors since there is no evidence available that shows a correlation between the two factors. Furthermore, the flavor and fragrance industry is a broad category and does not necessarily reflect the increase in sales of added flavors.

## Conclusions

The present opinion article analyzes the potential contribution of added flavors to excessive calorie intake and body weight gain in animals and humans. Added flavors are extensively tested concerning toxicity but no studies in humans exist examining the link between added flavors, food intake, and body weight gain. Therefore, only indirect evidence is available at present that added flavors might contribute to obesity in humans.

Based on the arguments presented in the current opinion article, the role of added flavors in human food intake regulation and body weight control needs to be assessed in future studies. More specifically, the following three knowledge gaps should be addressed within the next 10 years:

1. Differences in food intake between food items with added flavors and their non-flavored counterparts need to be assessed by consumer sensory evaluations and double-blind controlled trials in human volunteers.
2. The effectiveness of obesity interventions specifically targeting food items with added flavors should be elucidated within randomized controlled trials and compared to established treatments.
3. Independent associations between added flavor intake and relevant health outcomes including metabolic, as well as cardiovascular, morbidity, and mortality, should be defined in epidemiological cohorts.

Furthermore, it needs to be assessed how promotion of hedonic eating, disruption of flavor-nutrient learning, and other potential mechanisms might contribute to the overconsumption of flavored food and obesity risk in humans. In addition, policy strategies should be implemented which enable consumers to choose unflavored food items more easily. In most countries, added flavors are labeled on the ingredient list of packaged food [3, 92]. However, added flavors usually cannot be readily recognized in food prepared outside the home. Identification in this type of food is of importance since its consumption in the US has increased from 17% of average energy intake in 1977/1978 to 34% in 2011/2012 [93].

Addressing these gaps, outcomes, and policy strategies will better define the role of added flavors in human body weight control and potentially pave the way for novel, effective obesity treatment modalities which are urgently needed [94, 95].

## Supplementary Information

**Additional file 1. **Literature search.

**Additional file 2. **Studies on feed with added flavors (intervention) as compared to unflavored feed (control) in animal experiments.

**Additional file 3. **Obesity prevalence in the US and inflation-adjusted US flavor and fragrance industry sales since 1960.

## Data Availability

All data generated or analyzed during this study are included in this published article and its supplementary information file.

## Abbreviations

EAH: Eating in the absence of hunger
US: United States of America
