# Supplementary material for: Added flavors: potential contributors to body weight gain and obesity?
Source: BMC Med. 2022 Nov 1;20:417. doi: 10.1186/s12916-022-02619-3 (PMC9623908; doi:10.1186/s12916-022-02619-3)
Supplement: Supplementary file 1 — Additional file 1. Literature search. [file 12916_2022_2619_MOESM1_ESM.pdf]

Additional file 1. Literature search

The Opinion article is based on a literature search in PubMed, PMC, ScienceDirect, and Google Scholar up to September 1, 2021 with the following search terms or their combinations: “flavor”, “aroma”, “feed intake”, “hedonic eating”, “flavor-nutrient learning”, “body weight”, and “obesity”.

In addition to the keyword-based literature search, the following journals of the flavor and fragrance industry, as well as animal nutrition and livestock farming, were searched by hand (years in parentheses): Perfumery and Essential Oil Record (1951 to 1969), The Flavor Industry (1970 to 1974), International flavors and food additives: IFFA (1975 to 1979), Food: food ingredients & processing international (1980 to 1991), American Perfumer and Cosmetics (1959 to 1971), Perfumer and Flavorist international (1976 to 1980), Perfumer and Flavorist (1980 to 2017), FeedMagazine (2001 to 2020), Pig Progress (2016 to 2020), Dairy Global (2016 to 2020), All About Feed (2016 to 2020), World Poultry (2016 to 2020), Future Farming (2017 to 2020), including special editions. A further literature search was performed based on references cited in the articles found.

The literature was analyzed and structured by both authors. Inclusion criteria were as follows: Only studies with relevant obesity-associated endpoints including food/feed intake, body weight gain, and final body weight were included. In addition, the development of the flavor and fragrance industry including basic information about flavors, legal aspects, and sales, as well as time trends in obesity prevalence in humans and mechanistic studies concerning hedonic eating and flavor-nutrient learning were researched. Exclusion criteria were as follows: Research with a focus on phytogenic feed additives was excluded since they show various physiologic effects beyond flavoring of feed [8, 9]. Furthermore, studies on sweeteners were not included since their role in body weight control has been well summarized in recent reviews [10–13]. Animal studies elucidating the influence of flavors on feed intake and body weight were excluded if no adequate control group without flavor addition was present of if flavors were added combined with phytogenic feed additives and/or sweeteners.
