# Supplementary material for: Added flavors: potential contributors to body weight gain and obesity?
Source: BMC Med. 2022 Nov 1;20:417. doi: 10.1186/s12916-022-02619-3 (PMC9623908; doi:10.1186/s12916-022-02619-3)
Supplement: Supplementary file 2 — Additional file 2. Studies on feed with added flavors (intervention) as compared to unflavored feed (control) in animal experiments. [file 12916_2022_2619_MOESM2_ESM.pdf]

Additional file 2. Studies on feed with added flavors (intervention) as compared to unflavored feed (control) in animal experiments[1]

Study type	First author Year (Ref.)	Animals in I and C groups	Species	Period of life	Duration of I	Added flavor tested	Feed intake	Body weight gain	Final body weight	Further comments
1	De Rosa 2002 [41]	Same	Goat	2 to 3 y	6 d within 14 d	Clover, Ryegrass	↑[a]	n.p.	n.p.	[a]$p < 0.001$ for ryegrass in two of two sessions; $p < 0.05$ for clover in one of two sessions
	Khelil-Arfa 2021 [42]	Same[#]	Pony	4 to 13 y	5 d	Anis, Apple, Caramel, Raspberry	↑[a]	n.p.	n.p.	[a]$p < 0.05$ only for apple
	Harper 2016 [43]	Same[#]	Cattle	Lactation	6 d	Anise, Fenugreek, Honey, Molasses, Orange, Thyme, Vanilla	↔	n.p.	n.p.	Experiment not adequately controlled for strong effect of bin position and C feed not included on each day
	Seabolt 2010 [44]	Same	Pig	Post-weaning	2 d	Creamy and milky cheese profile with sweet and vanilla bottom notes (Luctarom®)	↓[a]	n.p.	n.p.	[a]$p < 0.01$
2	Thomas 2007 [45]	Same[†,#]	Cattle	Calves	21 d (3x7 d)	Orange, Vanilla added to water	↑[a]	↑[b]	n.p.	[a]$p < 0.05$; [b]$p < 0.05$ only for orange
		Same[†]		Second-lactation	28 d (4x7 d)	Orange added to water	↔	n.d.	n.p.	-
	Wene 1982 [46]	Same[†,#]	Baboon	7 to 15 y	48 d	Chocolate, Fruit punch, Lemon, Orange	(↑)[a]	n.d.	n.p.	[a]$p = 0.052$; The proportion of simple carbohydrates was different between flavored (25 %) and unflavored (5 %) chow which might bias the findings; Unflavored feed was also offered in I period
		Same[†,#]			48 d	Apple, Lemon, Orange, Sugar	↔	n.d.	n.p.	
		Same[†,#]			185 d	Orange, Punch	↑[a]	n.d.	n.p.	[a]$p = 0.034$
3	Danielsen 1991 [47]	Different	Pig	Pre-weaning	14 d	Cream, Strawberry	↑[a], (↑)[b]	↔	↑[c]	[a]$p < 0.05$ for cream; [b]$p \leq 0.1$ for strawberry; [c]p not given, 0.2 kg higher; [d]p not given,
				Post-weaning	21 d		↑[a], (↑)[b]	↑[a]	↑[d]	

				Entire period	35 d		↑[a], (↑)[b]	↑[a,e]	↑[d]	0.7 kg higher; [e]$p < 0.05$ for strawberry
	Yan 2011 [48]	Different	Pig	Pre-weaning	17 d	Cheese, Vanilla	↑[a]	↔	n.d.	[a]$p < 0.05$ only for cheese; [b]$p < 0.05$
				Post-weaning	7 d		↔	↑[b]	n.d.	
	Wang 2014 [49]	Different	Pig	Pre-weaning	21 d	Fruit-milk, Fruit-milk-anis	↑[a]	↔	↔	[a]$p < 0.05$ only for fruit-milk-anis; [b]$p < 0.01$ only for fruit-milk-anis
				Post-weaning	8 d		↑[b]	↑[b]	↔	
	Adeleye 2014 [50]	Different[#]	Pig	Pre-weaning	19 d	Apple, Apricot, Butterscotch, Red fruit, Toffee	↑[a]	n.d.	↔	[a]$p = 0.01$
				Post-weaning	14 d	-	↔	↑[a]	(↑)[b]	[a]$p = 0.03$; [b]$p = 0.07$
	King 1979 [51]	Different	Pig	Pre-weaning	27 d	Firanor	↔	↔	n.d.	[a]$p < 0.05$ only for piglets receiving flavored starter weaned from sows receiving flavored feed; [b]Same comparison as above, no specific p given but trend described in text
				Post-weaning	31 d		↑[a]	(↑)[b]	n.d.	
	Silva 2018 [52]	Different	Pig	Lactation	24 d	Krave® AP in sow feed (2 concentrations)	↑[a]	↔	↔	[a]$p < 0.001$
				Pre-weaning			n.d.	↑[a]	↑[b]	[a]$p < 0.001$ only for higher concentration; [b]$p < 0.001$
	Fathi 2009 [53]	Different	Cattle	Pre-weaning	~ 60 d	Vanilla	↑[a]	↑[b]	↑[a]	[a]$p = 0.03$; [b]$p = 0.01$; [c]$p = 0.02$
				Post-weaning	~ 21 d		↔	↔	↑[c]	
				Entire period	81 d		↑[c]	↔	↑[c]	
	Thomsen 1980 [54]	Different	Cattle	Pre-weaning	30 d	Butter, Maple , Milk	↔	↑[a]	n.d.	[a]$p < 0.05$ only for maple-flavored starter; [b]$p < 0.1$ only for maple-flavored starter; [c]$p < 0.1$ only for milk-flavored starter
				Post-weaning	21 d		(↑)[b]	(↑)[b]	n.d.	
				Entire period	51 d		(↑)[b]	↑[a], (↑)[c]	n.d.	
	Torrallardona 2000 [55]	Different	Pig	Post-weaning	35 d	Luctarom® (4 profiles)	↔	↑[a]	↑[a]	[a]$p = 0.01$ all profiles combined compared to C

3	Lv 2012 [56]	Different	Pig	95 d	14 d	Banana, Milk	↔	↑[a]	↔	[a]$p < 0.05$
	Dusel 2006 [57]	Different	Cattle	Calves	70 d	CuxArom Toffee Vanilla	↔[a]	↑[b]	(↑)[c]	[a]Intake of feed with added flavor only which was restricted from day 43 onwards; [b]$p = 0.045$; [c]$p = 0.078$
	Seabolt 2010 [44]	Different	Pig	Post-weaning	35 d	Creamy and milky cheese profile with sweet and vanilla bottom notes (Luctarom®)	↔	↔	↔	-
	McLaughlin 1983 [58]	Different	Pig	Post-weaning	35 d	Cheese, Sweet-molasses-caramel	↔	↔	n.d.	-
				Pre-weaning	8 d	Cheese, Commercial flavor, Sweet-molasses-caramel	n.d.	↔	↔	-
				Post-weaning	35 d		↔	↔	n.d.	
	Sulabo 2010 [59]	Different	Pig	Pre-weaning	4 d	Luctarom®	↔	↔	↔	-
				Post-weaning	28 d		↔	↔	↔	
	Danielsen 1981 [60]	Different	Pig	Pre-weaning	21 d	Suk-aroma	↔	n.d.	↔	-
				Post-weaning	35 d		↔	↔	↔	
	Naim 1985 [61]	Different[#]	Rat	n.d.	23 d	Bacon, Beef, Bread, Cheddar cheese, Cheese paste, Chicken, Chocolate, Liver, Nacho cheese, Peanut, Salami, Vanilla	↔	↔	n.d.	I group was exposed to both flavored and unflavored feed throughout the I period
	Seitz 2020 [62]	Different	Rat	n.d.	21 d	Peppermint	↔	↓[a]	n.d.	[a]$p < 0.05$ observed at three different flavor intensities

[1]Abbreviations used: C, Control; d, Days; I, Intervention; n.d., No data; n.p., Not possible due to study design; y, Years; ↑ indicates significant increase, ↓ significant decrease, ↔ no significant difference of intervention as compared to control, () trend, i.e., $p < 0.10$ and/or authors describe trend in text; [†]Intervention and control at different time points with same animals (i.e., within-subject design); [#]More than one added flavor was fed during one experimental setting to animals in the intervention group; Different animals refers to between-subject design; If a time range for the intervention was given, the shortest period was chosen as duration of intervention; Half days were rounded off; Start and end days of the intervention were counted as full days; Intervention groups with additional sweeteners or without added flavors were excluded from the table.
