# Supplementary material for: Added flavors: potential contributors to body weight gain and obesity?
Source: BMC Med. 2022 Nov 1;20:417. doi: 10.1186/s12916-022-02619-3 (PMC9623908; doi:10.1186/s12916-022-02619-3)
Supplement: Supplementary file 3 — Additional file 3. Obesity prevalence in the US and inflation-adjusted US flavor and fragrance industry sales since 1960. [file 12916_2022_2619_MOESM3_ESM.pdf]

Additional file 3. Obesity prevalence in the US and inflation-adjusted US flavor and fragrance industry sales since 1960[1]

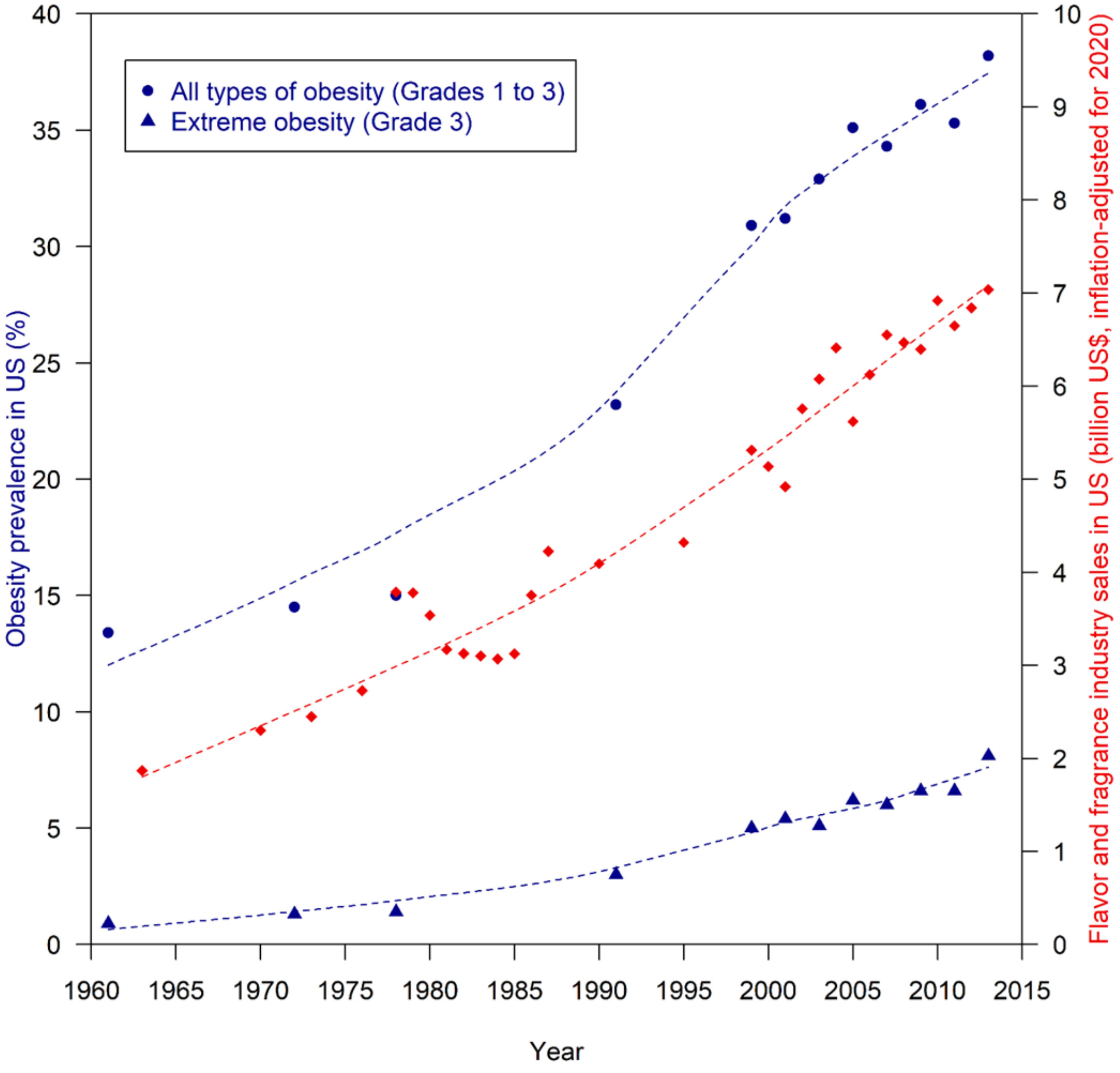

[1]Estimates for prevalence of obesity and extreme obesity are based on the National Health and Nutrition Examination Survey (NHANES) cohort, ages 20 to 74 years [91]. Years on the x-axis for NHANES data were assigned to the rounded off midpoint of the respective study period [91]. Estimates for sales of US flavor and fragrance industry are based on market figures [77–89]. In case of worldwide sales, US market share was calculated at 26.5 % using the average of three US market share data at different time points [80, 82, 83]. The sales in 1963 were calculated through the reported 6.6 % annual growth of the US flavor and fragrance industry from 1963 to 1973 based on sales in 1973 [77]. US flavor and fragrance industry sales are inflation-adjusted for 2020 US$ value [90]. Lines were fitted by locally estimated scatterplot smoothing.
